# Photophysiological response of Symbiodiniaceae single cells to temperature stress

**DOI:** 10.1038/s41396-022-01243-6

**Published:** 2022-04-26

**Authors:** Linhong Xiao, Sofia Johansson, Saskia Rughöft, Fabien Burki, Miguel Mendez Sandin, Maria Tenje, Lars Behrendt

**Affiliations:** 1 Department of Organismal Biology, Uppsala University, Norbyvägen 18A, SE-752 36 Uppsala, Sweden; 2 Department of Materials Science and Engineering, Science for Life Laboratory, Uppsala University, Box 35, SE-751 03 Uppsala, Sweden

**Keywords:** Environmental microbiology, Microbial ecology

## Abstract

Photosynthetic dinoflagellates in the family Symbiodiniaceae engage in symbiosis with scleractinian corals. As coral ‘bleaching’ is partly governed by the thermal sensitivity of different Symbiodiniaceae lineages, numerous studies have investigated their temperature sensitivity. However, the systematic identification of single-cells with increased temperature resistance among these dinoflagellates has remained inaccessible, mostly due to a lack of technologies operating at the microscale. Here, we employed a unique combination of microfluidics, miniaturized temperature control, and chlorophyll fluorometry to characterize the single-cell heterogeneity among five representative species within the Symbiodiniaceae family under temperature stress. We monitored single-cell maximum quantum yields (*F*_*v*_/*F*_*m*_) of photosystem (PS) II under increasing temperature stress (22‒39 °C, + 1 °C every 15 min), and detected a significant *F*_*v*_/*F*_*m*_ reduction at lineage-specific temperatures ranging from 28 °C to 34 °C alongside a 40- to 180- fold increase in intraspecific heterogeneity under elevated temperatures (>31 °C). We discovered that the initial *F*_*v*_/*F*_*m*_ of a cell could predict the same cell’s ability to perform PSII photochemistry under moderate temperature stress (<32 °C), suggesting its use as a proxy for measuring the thermal sensitivity among Symbiodiniaceae. In combination, our study highlights the heterogeneous thermal sensitivity among photosynthetic Symbiodiniaceae and adds critical resolution to our understanding of temperature-induced coral bleaching.

Ocean warming can disrupt the symbiosis between corals and photosynthetic dinoflagellates from the family Symbiodiniaceae, a process referred to as bleaching [1]. Corals can recover from bleaching by repopulating more stress-tolerant symbiont cells [2] and/or by recruiting new cells from the environment [3, 4]. The bleaching susceptibility of corals is thus partly linked to the thermal sensitivity of Symbiodiniaceae and many studies have experimentally tested lineage-specific thermal tolerances of coral symbionts [5–8]. However, the considerable heterogeneity between Symbiodiniaceae single cells remains underexplored [9], despite strong indications that the phenotypic richness among symbiont lineages is useful for mitigating bleaching effects [4, 10–13].

Here we studied the effect of temperature stress on Symbiodiniaceae single cells via microfluidics, microscale temperature control and pulse amplitude-modulated chlorophyll fluorometry (PAM) imaging. Specifically, we asked: (i) how heterogeneous are single-cells of Symbiodiniaceae under temperature stress and, (ii) does the initial photophysiology of a cell predict its thermal tolerance?

To address these questions, we selected five representative species from three Symbiodiniaceae lineages: *Effrenium* sp. (‘S1’, clade E, non-symbiotic), *Symbiodinium* sp. (‘S2’, ‘S3’ and ‘S4’, clade A, all symbiotic), and *Fugacium* sp. (‘S5’, clade F, symbiotic). The identity of all cell cultures was determined by phylogenetic analysis (Table S1 and Fig. S1) and their growth was inspected via absorbance measurements (Fig. S2). Following this characterization, single cells were loaded into individual microwells within a microfluidic device that was attached to a miniaturized temperature regulation system [14] and a PAM microscope (Fig. 1a‒c). Together, this setup enabled us to measure single-cell maximum quantum yields of PSII, i.e., *F*_*v*_/*F*_*m*_ = (*F*_*m*_ ‒ *F*_*0*_)/*F*_*m*_ under user-defined temperatures. As certain representative species (i.e., *Effrenium* sp. ‘S1’, *Fugacium* sp. ‘S5’) exhibited significantly reduced *F*_*v*_/*F*_*m*_ during stationary phase (Fig. S3), all experiments were conducted using exponentially growing cells.

**Fig. 1:**
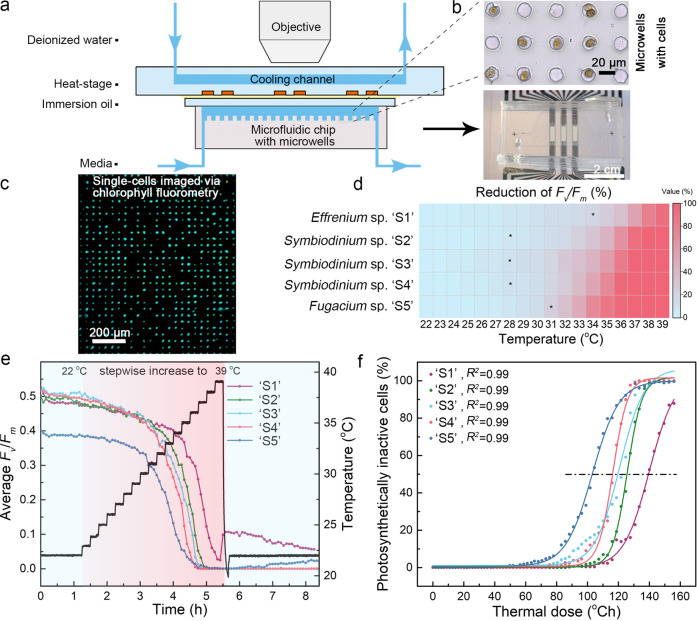
An integrated microfluidic approach to study the photophysiological responses of Symbiodiniaceae single cells under temperature stress. **a** The fully assembled temperature regulation system with the microfluidic chip attached using immersion oil, ready to be observed via a variable chlorophyll fluorescence imaging microscope (PAM). The temperature regulation system includes a heat-stage and a cooling channel (flushed with deionized water) and, in combination with PAM, enables single-cell measurements of *F*_*v*_*/F*_*m*_ under user-defined temperatures. **b** Top image, microwells with a diameter of 20 µm filled with individual cells of Symbiodiniaceae. Within the entire microfluidic device most microwells were empty (~54%) while the remaining microwells were filled either with single-cells (31%) or multiple cells (15%). Bottom image, the microfluidic device mounted onto the heat-stage. **c** False-color image of *F*_*v*_*/F*_*m*_ of Symbiodiniaceae single-cells immobilized within microwells of the microfluidic chip. **d** Heatmap depicting the reduction of average *F*_*v*_/*F*_*m*_ under stepwise increasing temperatures. Black stars denote the temperature at which *F*_*v*_/*F*_*m*_ values differed significantly, for the first time, when compared to *F*_*v*_/*F*_*m*_ values at 22°C (one-way ANOVA with *post hoc* Tukey tests, *p* < 0.05). **e** Average *F*_*v*_/*F*_*m*_ of five Symbiodiniaceae species exposed to stepwise increasing temperatures. In this profile, temperatures were increased by +1 °C every 15 min from 22 to 39 °C, followed by rapid cooling to 22°C for 2.5 h. Data was obtained from three individual experiments for each species (except for *Symbiodinium* sp. ‘S4’ where only two experiments were performed). The total number of single cells for each species were *n* = 784 for *Effrenium* sp. ‘S1’, *n* = 356 for *Symbiodinium* sp. ‘S2’, *n* = 313 for *Symbiodinium* sp. ‘S3’, *n* = 465 for *Symbiodinium* sp. ‘S4’, and *n* = 528 for *Fugacium* sp. ‘S5’. **f** Percentage of photosystem (PS) II inactive cells as a function of the thermal dose. Data points were fitted to dose–response curves to determine the half-maximal effective distress dose (*D*_*50%*_), i.e., the dose at which 50% cells did not maintain PSII activity. For this calculation, cells with *F*_*v*_/*F*_*m*_ values lower than 0.05 were considered PSII inactive. *D*_*50%*_ for each species is indicated in the figure using a horizontal black dashed line. See main text and supplementary materials for details on thermal dose calculation.

To understand the photophysiological heterogeneity among Symbiodiniaceae under temperature stress, we repeatedly measured single-cell *F*_*v*_/*F*_*m*_ under stepwise increasing temperatures (22‒39 °C, + 1°C every 15 min). This temperature range does not represent an environmentally realistic scenario but was chosen according to known growth conditions for coral symbionts [15] and in order to probe their thermal tolerance in a rapid assay (akin to [8], but using single cells). At cultivation temperature (22 °C), all five species demonstrated average *F*_*v*_/*F*_*m*_ ranging between 0.38‒0.53; however, under increasing temperatures, these values gradually declined in a lineage-specific manner (Fig. 1d, e). A significant (*p* < 0.05, one-way ANOVA with *post hoc* Tukey test) decline in average *F*_*v*_/*F*_*m*_ started to occur at 28 °C (‘S2’, ‘S3’, and ‘S4’), 31 °C (‘S5’), and 34°C (‘S1’) compared to their respective *F*_*v*_/*F*_*m*_ obtained at 22 °C (Table S2).

As stepwise increasing temperature exposes cells to aggregate stress, we calculated the cumulative thermal dose [14] (*D*) with a unit of °Ch via:

$$D = \int_{t_0}^{t_x} (T\left( t \right) - T_0)dt$$

where *t*_*0*_ represents the starting time in hours of the temperature profile, *t*_*x*_ represents the investigated time point in hours, *T(t)* and *T*_*0*_ (°C) represent the temperature at *t*_*x*_ and *t*_*0*_, respectively. By plotting the percentage of cells with inactive PSII as a function of *D* and fitting a dose-response curve (Fig. 1f), we determined the half-maximal effective distress dose (*D*_*50%*_) and the corresponding temperature where *D*_*50%*_ occurred. This revealed *D*_*50%*_ values of 140 °Ch for *Effrenium* sp. ‘S1’ (occurring at 37 °C), 126 °Ch, 121 °Ch, and 116 °Ch for *Symbiodinium* sp. ‘S2’, ‘S3’, and ‘S4’ (at 36 °C, 35 °C, and 35 °C, respectively), and 103 °Ch for *Fugacium* sp. ‘S5’ (at 33 °C). Single cells of Symbiodiniaceae thus respond differently to cumulative short-term temperature stress and certain species (e.g., *Effrenium* sp.) appear more temperature resilient than others (e.g., *Fugacium* sp.). These findings are broadly consistent with earlier studies on coral symbionts, where *F*_*v*_/*F*_*m*_ typically decreased above 31 °C [16–18].

To describe the phenotypic heterogeneity among Symbiodiniaceae single cells under temperature stress, we calculated a measure of photophysiological heterogeneity, *H* by [19]:

$$H = \frac{{std(F_v/F_m)}}{{\widetilde {F_v/F_m}}}$$

where $\widetilde {F_v/F_m}$ represents the average *F*_*v*_/*F*_*m*_ at a specific temperature and $std\left( {F_v/F_m} \right)$ represents the corresponding standard deviation. All species demonstrated increasing *H* values under elevated temperatures, but significantly higher *H* after exceeding the temperature where *D*_*50%*_ occurred (Fig. 2a, b). Maximal *H* values were 2.46 (‘S1’), 16.3 (‘S2’), 8.3 (‘S3’), 12.4 (‘S4’), and 12.8 (‘S5’) (Table S3 for all *H* values). Applying a thermal dose above *D*_*50%*_ thus increases the photophysiological heterogeneity among single cells. Notably, mid-exponentially growing cells exhibited lower *H* values compared to cells grown at stationary phase (Table S3), which suggests that the single cell heterogeneity also increases with culture age or, alternatively, due to other factors that change with time (e.g., microenvironments). In other microorganisms, single cell heterogeneity occurs due to stochastic gene expression or molecular-level ‘noise’ which increases the probability of specific phenotypes to persist in challenging environments [20]. Similar mechanisms could be in play for coral symbionts and our platform, in combination with single-cell selection [9] and sequencing methods, is uniquely suited to explore the molecular underpinnings of this heterogeneity and to accelerate targeted phenotyping efforts.

**Fig. 2:**
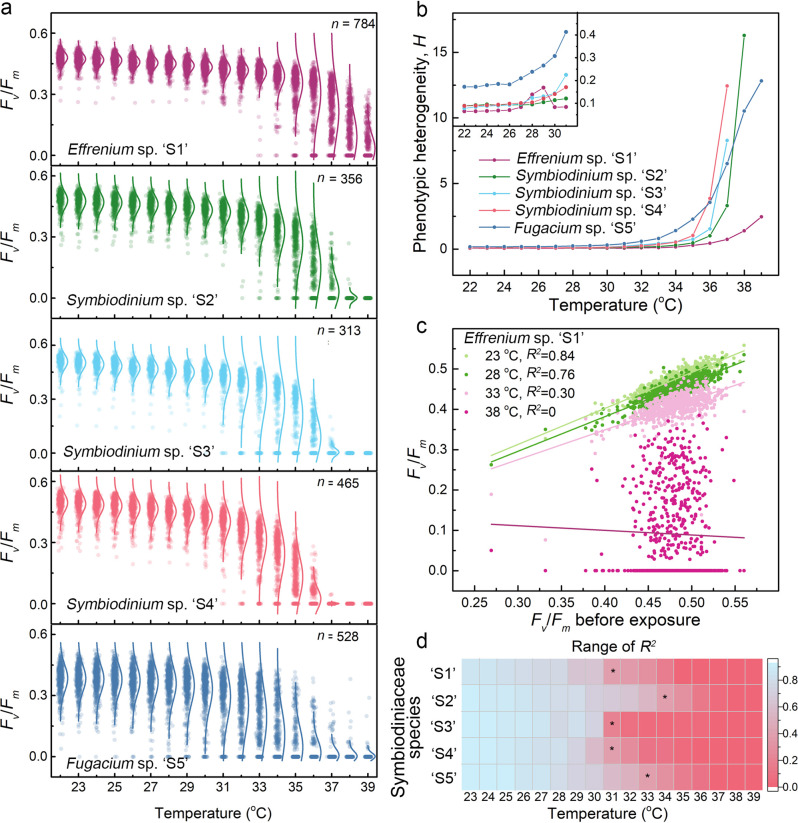
The photophysiological heterogeneity of Symbiodiniaceae single cells under elevated temperatures. **a** Single cell *F*_*v*_/*F*_*m*_ values from all investigated Symbiodiniaceae species under stepwise increasing temperatures. *F*_*v*_/*F*_*m*_ values at a given temperature were obtained by averaging three *F*_*v*_/*F*_*m*_ measurements performed at each temperature step for each cell (see supplementary materials and methods for details). Solid lines along scatterplots represent the fitting results to a normal distribution. **b** The phenotypic heterogeneity, *H*, of individual cells from five Symbiodiniaceae species under stepwise increasing temperatures. See main text and supplementary materials for details on the calculation of *H*. Inset: enlarged curves displaying *H* at 22‒31 °C. **c** Examples of correlations between *F*_*v*_/*F*_*m*_ before and after temperature exposure for single cells of *Effrenium* sp. ‘S1’. Here the *X*-axis represents the *F*_*v*_/*F*_*m*_ value from each individual cell before temperature exposure (at 22 °C) and the *Y*-axis represents the *F*_*v*_/*F*_*m*_ value from the same cell under stepwise increasing temperatures. Note that only data on four temperature conditions (23, 28, 33, and 38 °C) are displayed to increase overall readability of the figure. Linear regression fittings and corresponding *R*^*2*^ values are shown in the legend. **d** Heatmap of *R*^*2*^ values for single cells of five Symbiodiniaceae species at each temperature. *R*^*2*^ values were obtained from linear regressions of scatterplots between single cell *F*_*v*_/*F*_*m*_ values at elevated temperatures and *F*_*v*_/*F*_*m*_ values of the same cell at 22 °C (see **c** for example correlations). Black stars denote the temperature at which the linearity starts to collapse (i.e., *R*^*2*^ < 0.5).

We explored whether the innate photophysiology of a cell could reflect its ability to resist future temperature stress by plotting the initial *F*_*v*_/*F*_*m*_ (at 22 °C) of a cell against its *F*_*v*_/*F*_*m*_ at increasing temperatures and linear regression fitting (Fig. 2c and Fig. S4‒S8). This revealed a linear correlation (*R*^*2*^ > 0.5) at temperatures below 31 °C for all species and a collapse of this linearity (*R*^*2*^ < 0.5) for three out of five species above 31°C (Fig. 2d). This suggests that the initial photophysiology of a cell at 22 °C is useful in predicting its PSII activity at temperatures up to 31 °C. Our inability to predict PSII activity above 31 °C is likely related to the simultaneous increase in phenotypic heterogeneity beyond this temperature. Despite these observed trends we cannot rule out that Symbiodiniaceae cells with low initial *F*_*v*_*/F*_*m*_ could still exhibit high thermal tolerance. For example, photoacclimation can result in lower *F*_*v*_*/F*_*m*_ while cells maintain thermal tolerance [17]. Conceivably, non-ideal cultivation temperatures could also have led to the reduction of initial *F*_*v*_*/F*_*m*_ values among our cultures and so could have microenvironmental gradients within cultivation vessels (e.g., small differences in irradiance or gas transfers due to stratified growth of cells). Despite these potential shortcomings, earlier work corroborated that the application of acute heat stress to corals could resolve fine differences in host thermal tolerance [8]. We therefore speculate that our minimally-invasive method holds potential to provide bottom-up information on the thermal sensitivity of corals but also emphasize that further experimental verification is needed.

In summary, our study (i) uncovered increasing levels of single cell heterogeneity under elevated temperatures and (ii) reliably predicted photophysiological responses of single cells to temperatures below 31 °C. Finally, besides temperature, our approach can also be used to reproduce other environmental features experienced by symbionts *in hospite* (e.g., pH and nutrients) and thus help elucidate the interplay between temperature stress, chemical microenvironment and acidification on coral symbionts.

## Supplementary information

Supplementary information
