# Supplementary material for: Photophysiological response of Symbiodiniaceae single cells to temperature stress
Source: ISME J. 2022 Apr 26;16(8):2060–4. doi: 10.1038/s41396-022-01243-6 (PMC9296599; doi:10.1038/s41396-022-01243-6)
Supplement: Supplementary file 1 — Supplementary information [file 41396_2022_1243_MOESM1_ESM.pdf]

Supplementary information

Photophysiological response of Symbiodiniaceae single cells to temperature stress

Linhong Xiao[1], Sofia Johansson[2], Saskia Rughöft[1], Fabien Burki[1], Miguel Mendez Sandin[1], Maria Tenje[2] and Lars Behrendt[1,*]

[1]Department of Organismal Biology, Uppsala University, Norbyvägen 18A, SE-752 36 Uppsala, Sweden.

[2]Department of Materials Science and Engineering, Science for Life Laboratory, Uppsala University, Box 35, SE-751 03 Uppsala, Sweden.

[*]Corresponding author E-mail: lars.behrendt@scilifelab.uu.se

Keywords:

Temperature stress, Symbiodiniaceae, photophysiological response, single-cells, microfluidics.

This document includes:

- Supplementary Materials and Methods
- Supplementary Tables S1 to S3
- Supplementary Figures S1 to S8
- Supplementary references

Supplementary Materials and Methods

Materials. SYLGARD™ 184 silicone elastomer kits were purchased from the Dow Chemical Company. Symbiodiniaceae cultures *Effrenium* sp., *Fugacium* sp., and *Symbiodinium* sp. were purchased from the Bigelow culture collection (Table S1 and Figure S1 for detailed information). f/2 medium was prepared with a salinity of 36 (ppt) by adding the following components: $NaNO_3$ (75 g L^{-1}, 1 mL), NaH_2PO_4 H_2O (5 g L^{-1}, 1 mL), $Na_2SiO_3 \cdot 9H_2O$ (30 g L^{-1}, 1 mL), trace metal solution (1 mL), and vitamin solution (0.5 mL) to 1 L of autoclaved and sterile-filtered (bottle-top vacuum filter, 0.2 µm, Corning, USA) artificial seawater (Instant Ocean, Aquarium Systems, Sarrebourg, France).

Fabrication of microfluidic devices. Microfluidic devices were constructed using standard photolithography techniques by following previously reported procedures[1]. In brief, a master mold was constructed in clean-room facilities using a clean 4" Si wafer. A resist layer of SU 8-2100 (70 µm) was spin coated on the wafer to form flow channels, inlets and outlets after curing with UV exposure. On top of this first layer, another resist layer of SU 8-2015 (15 µm) was spin coated to form the cell compartments (i.e., microwells). After obtaining the master mold, PDMS prepolymer was prepared by mixing elastomer monomer and curing agent at a ratio of 10:1 by weight, and degassed in a vacuum chamber to completely remove air bubbles. The PDMS prepolymer was then carefully poured on the master mold and baked at 80°C for 24 h. Afterward, the PDMS layer was manually removed from the master mold with a scalpel and cut into individual microfluidic chips. Chip surfaces were covered with Scotch tape and holes were punched with a 1.5 mm-diameter biopsy puncher (World Precision Instruments, Sarasota, FL, USA) to obtain inlets and outlets. To bond microfluidic devices, PDMS layers and microscopy cover slips (25 × 60 mm, 170 µm thickness) were exposed to oxygen plasma for 1 min (Zepto 1, Diener electronics GmbH, Ebhausen, Germany) and placed overnight on a heating plate at 80°C. Microfluidic chips were kept covered with Scotch tape in a Petri dish until use.

Fabrication of the temperature regulation system. The temperature regulation system was assembled according to previously reported procedures[1]. It consists of four parts: (i) a heat-stage with an integrated array of six individual Joule heaters and resistance temperature detectors (RTDs), (ii) a temperature controller to regulate the power applied to the Joule heaters, (iii) a liquid cooling system

to improve the thermal stability, and (iv) a GUI software program to control other parts to obtain desired temperature profiles. The Joule heaters and RTDs were made of Ni by using UV lithography technologies, and the obtained thin-film Joule heaters and RTDs have resistances of 65–70 Ω and 60–65 Ω, respectively. The temperature of the system was regulated via the temperature-controlling unit and the software in automatic mode. The assembled system has three optically transparent windows (6.0 mm × 8.5 mm for each), which matches the microwell channels of the microfluidic chip and allows for pulse amplitude-modulated chlorophyll fluorometry (PAM) microscopy observation of immobilized cells.

Cell culture conditions. Cultures of five non-axenic Symbiodiniaceae species were grown without the addition of any antimicrobial control agents during experiments or standard maintenance. All cultures were maintained in f/2 medium at a salinity of 36 PSU and a pH of 8.0 in Nunclon EasYFlasks (culture area: 25 cm^2, Thermo Fisher Scientific, Waltham, MA, USA) at 22°C over a 14 h/10 h day/night cycle without shaking. Irradiance was provided by white light-emitting diodes with a photon irradiance (400 to 700 nm) of about 100 μmol photons m^{-2} s^{-1} in a temperature-controlled incubator (AlgaeTron AG230, Photon Systems Instruments, Czech Republic). Irradiance was quantified by inserting a submersible spherical micro quantum sensor (US-SQS/L, Walz, Effeltrich, Germany) connected to a universal light meter (ULM-500, Walz, Effeltrich, Germany) into a cultivation vessel filled with f/2 medium. To obtain the growth curve of all species, we measured the optical density (OD) of Symbiodiniaceae cell populations in 24-well plates over time. Cultures were diluted into fresh f/2 medium so that the initial OD was 0.004 after subtracting the OD of medium. Then, cells were inoculated into 1 mL f/2 medium in a 24-well flat-bottom plate with four wells in one plate for each strain and four wells containing only f/2 medium which were measured as the control group (background OD). Three technical replicates were measured for all five species. All 24-well plates were kept in the incubator except during OD measurements. The OD was determined as absorbance at 680 nm and was measured after shaking for 5 s in a microplate reader (TECAN Spark, Tecan Trading AG, Switzerland) every two days, until cultures had reached late stationary phase. The obtained growth curves are shown in Figure S2 and, for experiments, cells were inoculated in fresh f/2 medium at a ratio of 1:100 (v/v) in order to obtain exponentially growing cells during experiments.

Cell loading. Exponentially growing cells (10 to 14 days after inoculation) were mixed thoroughly by pipetting the culture suspension and 6 mL of this culture suspension was centrifuged for 2 min at 1500 rpm. The supernatant was removed and cells were re-suspended in 50 µL fresh f/2 medium. The resulting concentrated cell suspension was re-suspended via pipetting and then injected into the inlet of the pre-vacuumed microfluidic chip. This resulted in microwells which were visibly overlaid with cells. Following cell injection, the flexible PDMS channels were repeatedly compressed using the blunt end of a pair of forceps to immobilize single cells into microwells. This immobilization procedure was previously found to not affect the photophysiology of Symbiodiniaceae cells[2]. Following cell immobilization, fresh f/2 medium was gently flushed into the device to wash out any remaining non-immobilized cells. The resulting microfluidic device was covered with Scotch tape and placed in the incubator for 1~2 h before use. Using this procedure, we achieved a loading efficiency between 15% (doublets/triplets) and 31% (single-cells). The remaining 54% of microwells remained empty. To ensure that only single-cell data was used in data analysis, single-cells were selected manually using the ImagingWin software (see PAM imaging for details).

Thermal exposure of Symbiodiniaceae. Thermal exposure experiments were performed using a microfluidic device coupled to a temperature regulation system according to previously reported procedures[1]. Briefly, prior to thermal exposure the temperature regulation system was calibrated using the reference chip (a microfluidic chip with an embedded reference Pt-100 temperature sensor, IEC751 Class A, 666-7359 RS PRO). Here, the temperature of the system was elevated stepwise from 22 to 39°C with a duration of 75 min at 22°C and 15 min at each other temperature, followed by a ramp down to 22°C. During this process, the acquired temperature profile from the reference temperature sensor was recorded as a calibration file, which was used to calibrate the temperature regulation system. The calibration procedure was performed in the beginning of every week before experiments were conducted.

For thermal exposure experiments, the microfluidic chip was carefully attached to the heat-stage by applying one droplet of immersion oil (Immersol 518N, Carl Zeiss) between them. The channels containing microwells were aligned with the optically transparent windows of the heat-stage using a stereomicroscope (SMZ800N, Nikon, Japan) and then affixed using Scotch tape. Afterwards, the fully assembled temperature regulation system and microfluidic chip was mounted onto a PAM microscope

to observe the photosynthetic activity of single-cells under temperature stress. During experiments, cells were provided with a continuous flow of fresh f/2 medium via a glass syringe (25 ml, Hamilton 1000 series, Hamilton Corp.) mounted onto a syringe pump (neMESYS, Cetoni GmbH, Korbussen, Germany). The flow rate was set as 800 μL h^{-1}. The flow of DI water in the cooling channel of the temperature regulation system was set at a rate of 60 μL min^{-1} using a pressure pump (OB1 MK3+, ElveFlow, France). After dark adaptation for 15–20 min, exponentially growing (10–14 days old) Symbiodiniaceae cells were exposed to stepwise elevated temperatures from 22 to 39°C with a duration of 75 min at 22°C to allow for thermal equilibration of cells and 15 min at each other temperature. Thermal exposure experiments were replicated three times for each species over the course of 2 months, except for *Symbiodinium* sp. 'S4' where only two experiments were performed.

PAM imaging. A multicolor variable PAM imaging microscope (IMAG-RGB; Heinz Walz GmbH, Effeltrich, Germany) was used to measure the photosynthetic efficiency of Symbiodiniaceae single-cells using a 10× objective (Zeiss Fluar 10×/ 0.5). The maximal quantum yield of photosynthesis in photosystem II, i.e., $F_v/F_m = (F_m - F_0)/F_m$ within single-cells was measured by applying a saturation pulse every five minutes after the initial dark adaptation. Here, F_v, F_m, and F_0 represent the variable, maximal, and minimal fluorescence of dark-adapted cells, respectively. The intensity of the saturation pulse light was optimized for each species to ensure that this acquisition frequency did not cause a significant reduction of F_v/F_m during thermal exposure experiments (Supplementary Fig. S4–S8). In all our experiments, cells were dark adapted for 15 to 20 min at 22°C before the temperature was increased. PAM images were captured using the supplied ImagingWin PAM software (Walz GmbH, Germany). A more detailed description about the software and the PAM system can be found in previous articles[1,2].

To investigate the effect of growth state on photosynthetic functioning, we measured the F_v/F_m of single-cells from all five species upon reaching mid-exponential and stationary growth (about 100 hours after the exponential phases ended). The resulting data are shown in Figure S3.

Phylogenetic identification of Symbiodiniaceae species. Between 100 – 200 mg of pelleted culture material was frozen in liquid nitrogen and ground to a fine powder. From this material, DNA was extracted using the Wizard Genomic DNA Purification Kit (Promega Biotech AB; Nacka,

Sweden) according to the manufacturer's instructions for DNA isolation from plant tissue. Amplification of the LSU region was performed according to LaJeunesse *et al.*[3] using the primers 28S-forward (5`-CCCGCTGAATTTAAGCATATAAGTAAGCGG-3`) and 28S-reverse (5`-GTTAGACTCCTTGGTCCGTGTTTCAAGA-3`). PCR reactions were set up as 25 µl reactions using the KAPA HiFi HotStart ReadyMix (Roche AB, Solna, Sweden) master mix and the following thermocycler conditions: 3 min at 90°C, followed by 35 cycles of 20 sec at 98°C, 20 sec at 65°C, and 20 sec at 72°C, and then 5 min at 72°C. PCR products were sent to Macrogen Europe B.V. (Amsterdam, The Netherlands) for purification and sequencing. After sequencing, forward and reverse sequences were checked and assembled using ChromasPro software version 2.1.10[4]. Assembled sequences were aligned against the reference 28S ribosomal DNA (rDNA) alignment detailed in LaJeunesse *et al.*[3] (downloaded from the Dryad repository: doi.org/10.5061/dryad.1717129) with the '--add' option from the mafft v7.453 package[5]. Ambiguous positions were not removed[6]. The final dataset, containing 84 taxa and 732 positions, was used to infer phylogenetic analysis by RAxML[7] under the GTR+Gamma evolutionary model with 1000 rapid bootstraps. Final tree was visualized and edited with FigTree version 1.4.3[8]. All sequences obtained in this study were submitted to GenBank and are publicly available via the following accession numbers: OM337795-OM337799.

Data analysis. Single-cell data were selected manually within the PAM software. The resulting single-cell F_v/F_m data were analyzed using OriginPro 2016 (b9.3.226, OriginLab Corporation, Northampton, MA, USA). The reduction of average F_v/F_m at elevated temperatures was calculated as $Reduction\ (\%) = \frac{\Delta(F_v/F_m)_T}{(F_v/F_m)_{22}} \times 100\%$, where $(F_v/F_m)_{22}$ represents the average F_v/F_m values of all single-cells from replicates for each species at 22°C, and $\Delta(F_v/F_m)_T$ represents the difference between average F_v/F_m at a given temperature and that at 22°C. Average F_v/F_m values at a given temperature were obtained by averaging the three F_v/F_m measurements performed at each temperature step for each cell. The thermal dose was calculated from[1]: $D = \int_{t_0}^{t_x} (T(t) - T_0)dt$, where D represents the thermal dose, t_0 represents the starting time of the temperature profile, t_x represents the investigated time point, $T(t)$ represents the temperature at t_x and T_0 represents the cultivation temperature of cells (= 22°C). A measure of photophysiological heterogeneity, H was calculated by[9]:

$H = \frac{std(F_v/F_m)}{\widetilde{F_v/F_m}}$ where $\widetilde{F_v/F_m}$ represents the average F_v/F_m value of cells at a specific temperature and $std(F_v/F_m)$ represents the corresponding standard deviation. Linear regressions were performed using the "simple fit" application within Origin and adjusted R^2 values are displayed. Non-linear regressions were performed using the "Dose Response" function within Origin, and adjusted R^2 values are displayed. Growth curve fitting were performed using the "sigmoidal logistic" function within Origin. Significance levels were calculated using one-way ANOVA with *post hoc* Tukey tests to assess differences between different temperature treatments, with $p < 0.05$ considered to indicate statistical significance of detected differences.

Supplementary Tables

Table S1. Description of the five Symbiodiniaceae species used within this study. Cultures were ordered from the National Center for Marine Algae and Microbiota at Bigelow Laboratory (NCMA) and phylogenetic information obtained via in-house 18S *rDNA* sequencing (see supplementary materials and methods). Symbiodiniaceae lineage assignments, hosts, origins and lifestyle are also provided.

Culture ID	Source	Clade/ lineage	Symbiodiniaceae species	Host	Origin	Lifestyle
S1	CCMP421	E	*Effrenium* sp.	–	Cook Strait	Free-living
S2	CCMP2459	A	*Symbiodinium* sp.	*Oculina diffusa*	Sargasso Sea	Symbiotic
S3	CCMP2464	A	*Symbiodinium* sp.	*Cassiopeia xamachana*	Florida (Caribbean Sea)	Symbiotic
S4	CCMP2467	A	*Symbiodinium* sp.	*Stylophora pistillata*	Gulf of Aqaba	Symbiotic
S5	CCMP2455	F	*Fugacium* sp.	*Meandrina meandrites*	Jamaica (Caribbean Sea)	Symbiotic

Table S2. Significance levels of the differences between average F_v/F_m values at 22°C and F_v/F_m values under increasing temperatures. Here shown are the temperatures at which F_v/F_m values declined significantly for the first time compared to F_v/F_m values at 22°C. Significance was determined via a one-way ANOVA analysis in combination with a *post hoc* Tukey test.

Culture ID	Temperature (ºC)	df	Sum of squares	F value	*p*-value
Effrenium sp. 'S1'	22°C vs. 34°C	1	3.19	1889.40	<0.05
Symbiodinium sp. 'S2'	22°C vs. 28°C	1	0.19	113.24	<0.05
Symbiodinium sp. 'S3'	22°C vs. 28°C	1	0.46	268.30	<0.05
Symbiodinium sp. 'S4'	22°C vs. 28°C	1	0.44	236.09	<0.05
Fugacium sp. 'S5'	22°C vs. 31°C	1	2.13	239.08	<0.05

Table S3. The photophysiological heterogeneity, *H*, of individual cells of five Symbiodiniaceae species under stepwise increasing temperatures. *H* was calculated by dividing the standard deviation of the average F_v/F_m value at a certain temperature by their corresponding average F_v/F_m at the same temperature. As a control, *H* of individual cells at 22°C at mid-exponential growth or stationary growth were also calculated.

Temperature	Photophysiological heterogeneity, *H*				
	Effrenium sp. 'S1'	*Symbiodinium* sp. 'S2'	*Symbiodinium* sp. 'S3'	*Symbiodinium* sp. 'S4'	*Fugacium* sp. 'S5'
22°C mid-exponential	0.12	0.15	0.13	0.17	0.13
22°C stationary	0.32	0.20	0.22	0.16	0.24
22°C	0.06	0.09	0.08	0.09	0.17
23°C	0.06	0.09	0.08	0.09	0.17
24°C	0.06	0.10	0.09	0.09	0.18
25°C	0.06	0.09	0.09	0.09	0.19
26°C	0.06	0.09	0.09	0.10	0.18
27°C	0.07	0.09	0.10	0.10	0.21
28°C	0.07	0.09	0.12	0.10	0.24
29°C	0.07	0.10	0.13	0.12	0.27
30°C	0.07	0.11	0.14	0.14	0.31
31°C	0.07	0.12	0.22	0.17	0.41
32°C	0.10	0.16	0.32	0.24	0.59
33°C	0.11	0.20	0.44	0.36	0.81
34°C	0.16	0.29	0.55	0.52	1.4
35°C	0.28	0.46	0.73	1.03	2.27
36°C	0.44	1.01	1.54	3.87	3.55
37°C	0.74	3.30	8.28	12.43	6.51

38°C	1.39	16.28	–*	–*	10.52
39°C	2.46	–*	–*	–*	12.82

* Cells from *Symbiodinium* sp. ‘S2’, *Symbiodinium* sp. ‘S3’, *Symbiodinium* sp. ‘S4’ did not show *H* values at 38 and 39°C as the F_v/F_m of cells could not be measured at these temperatures.

Supplementary Figures

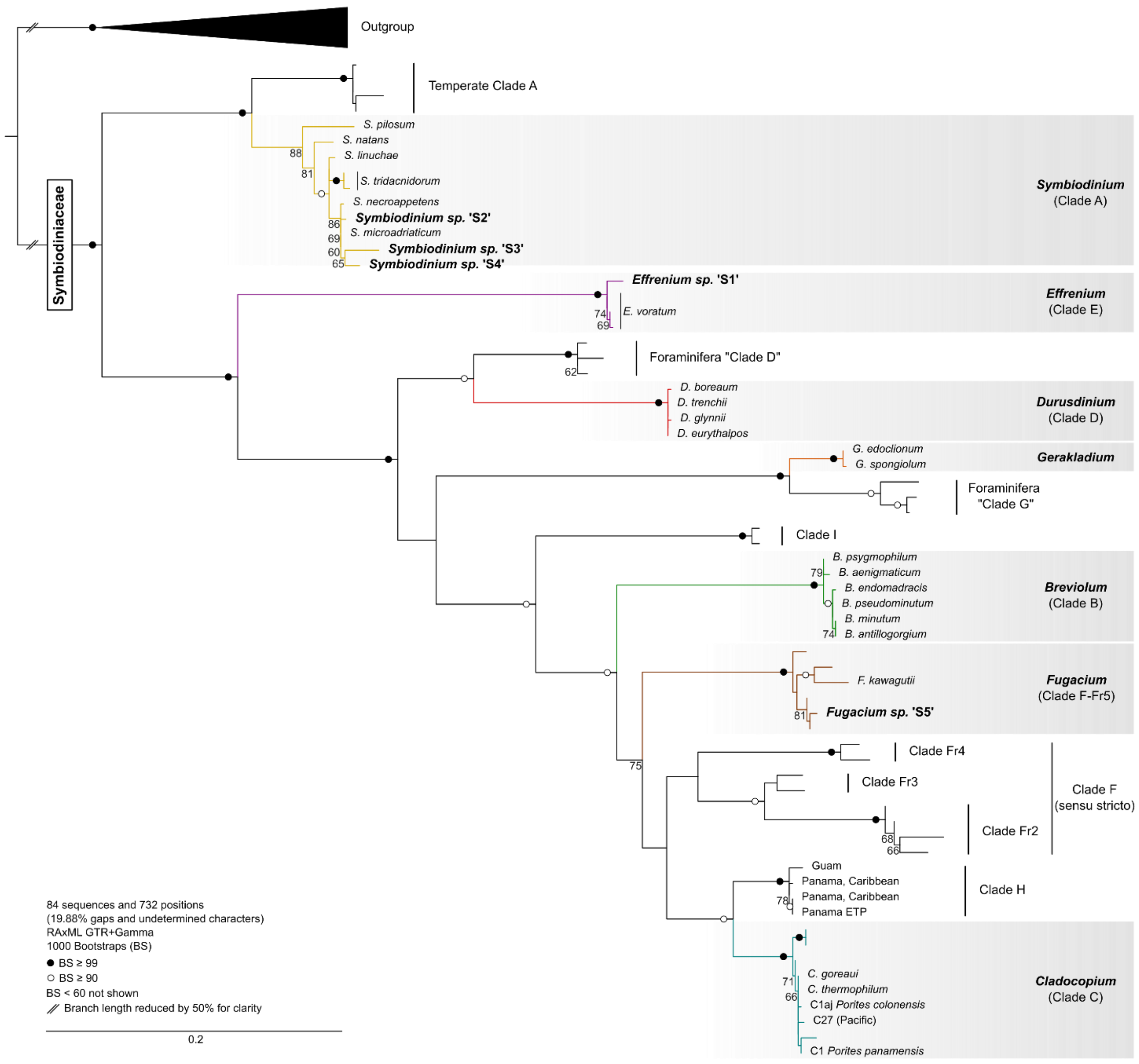

Figure S1. Phylogenetic position of the cultures used in this study within the family Symbiodiniaceae. Phylogeny was inferred from the partial 28S rDNA (LSU; D1-D2 regions) with 84 taxa and 732 aligned positions (within the framework of LaJeunesse *et al.*[3]). The tree was obtained with Maximum

Likelihood implemented in RAxML using the GTR + Gamma model of sequence evolution, with 1000 rapid bootstrap replicates for measuring support. Black circles indicate BS ≥ 99%; hollow circles indicate BS ≥ 90%; BS < 60 are not shown. Sequences obtained in this study are shown in bold. Nineteen sequences within Dinophyceae were assembled as outgroup (for further details, please see LaJeunesse *et al.*[3]). Branches with a double barred symbol are twofold reduced for clarity.

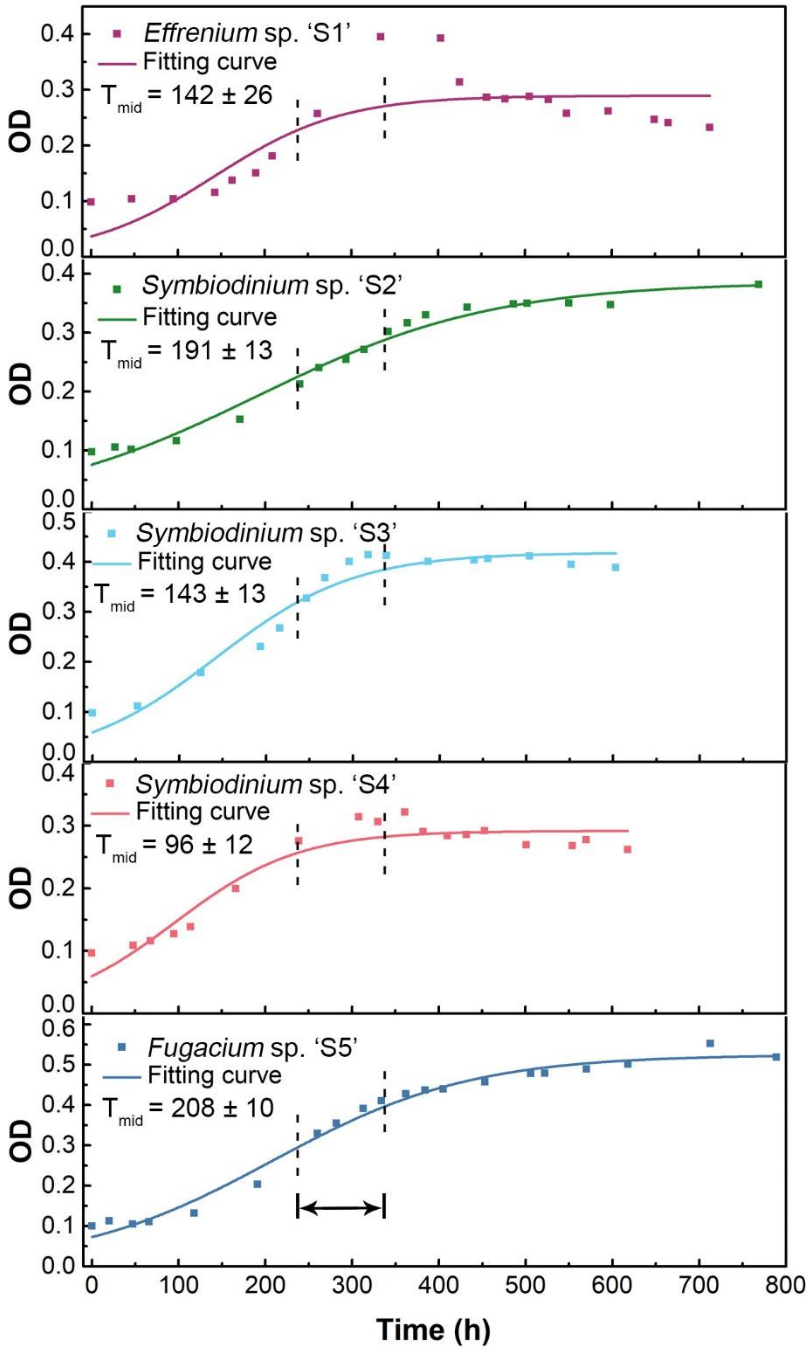

Figure S2. Growth curves of all investigated Symbiodiniaceae species. Solid lines represent the

fitting results to a sigmoidal logistic function. T_{mid} (with a unit of hours) represents the timepoint when cells reached mid-exponential growth. Dark dashed lines represent the time at which three independent temperature exposure experiments were conducted (10–14 days post-inoculation).

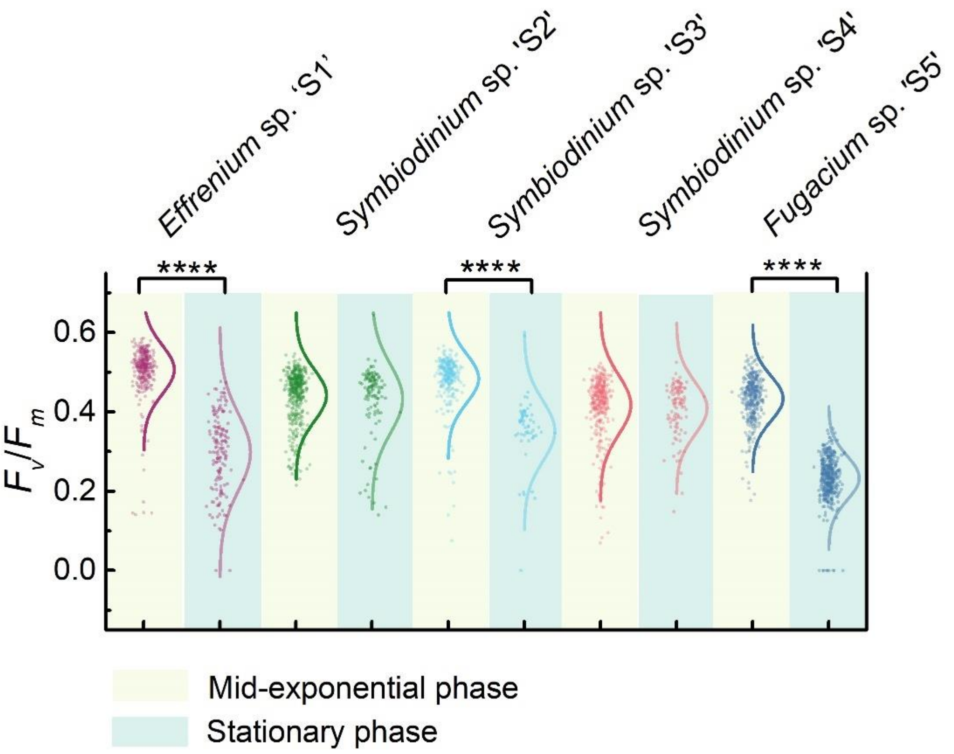

Figure S3. **The effect of growth phase on the single-cell photophysiology of five Symbiodiniaceae species**. Single cell F_v/F_m values from five Symbiodiniaceae species were obtained either for cells growing midexponentially (yellow background) or at their stationary phase (blue background, about 100 hours after the exponential phases ended), respectively. Solid lines along scatterplots represent the fitting results to a normal distribution. Significance levels were calculated using one-way ANOVA with *post hoc* Tukey tests to assess differences between the distribution of single cell F_v/F_m values between growth states, with $p < 0.05$ considered to indicate statistical significance of detected differences. Asterisks denote significant differences (**** = $p < 0.0001$).

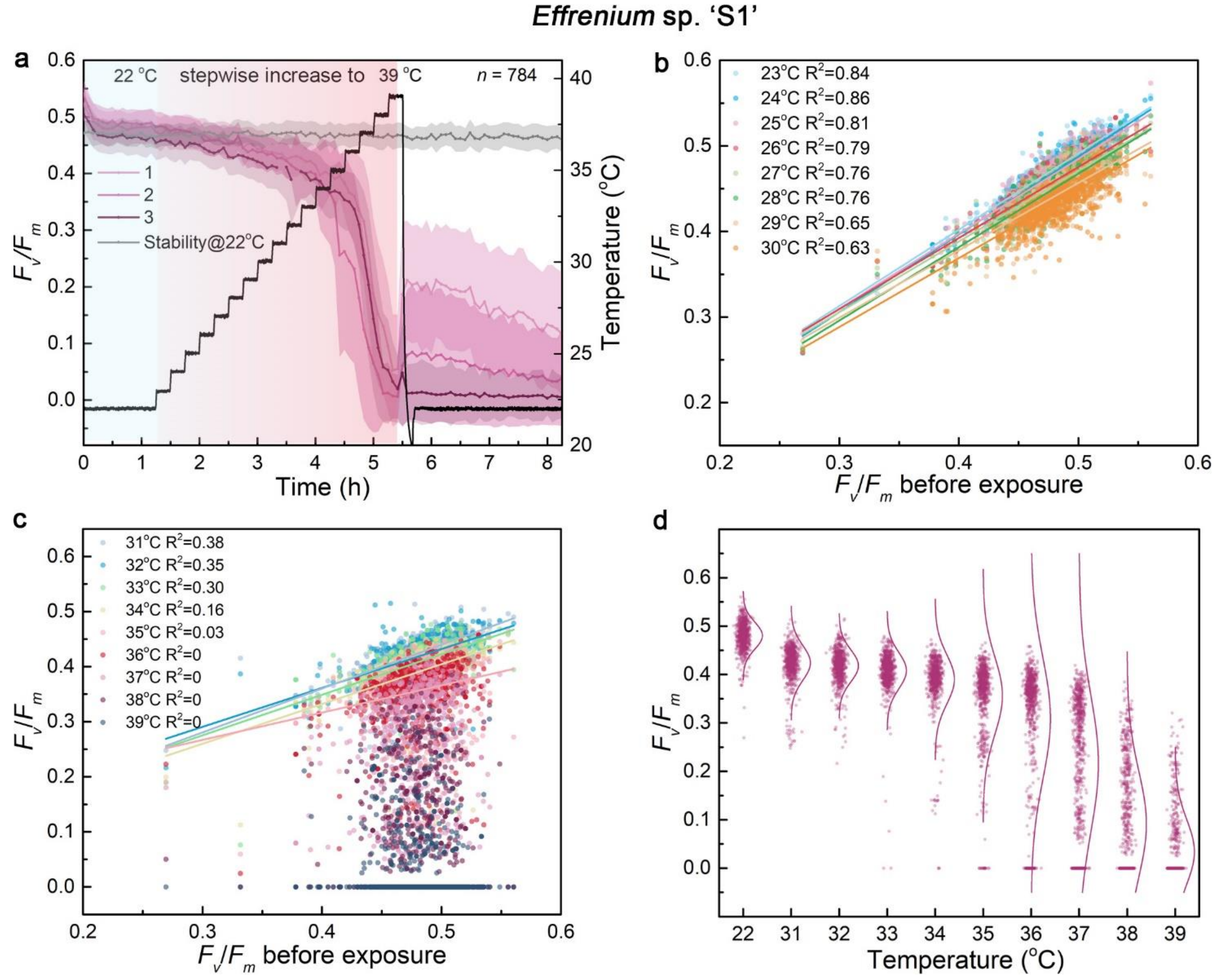

Figure S4. The photophysiological response of *Effrenium* sp. 'S1' single-cells to elevated temperatures. (a) The inter-experimental variability of average F_v/F_m under stepwise increasing temperatures. Average F_v/F_m values were calculated from single-cells from three independent biological experiments. These experiments (colored lines) describe the reduction in F_v/F_m under stepwise increasing temperatures (black line) and an additional experiment (gray line) that investigated the stability of F_v/F_m under repeated saturation probing but without temperature exposure. Shaded areas represent the standard deviation in each experiment. (b) Correlations between F_v/F_m before and after temperature exposure for single-cells of *Effrenium* sp. 'S1'. Here, the X-axis represents the F_v/F_m value from each individual cell before temperature exposure (at 22°C) and the Y-axis represents the F_v/F_m value from the same cell under stepwise increasing temperatures (from 23 to 30°C). F_v/F_m values at a given temperature were obtained by averaging the three F_v/F_m measurements performed at each temperature step for each cell (see supplementary materials and

methods for details). Linear regression fits of the resulting scatterplots are shown together with their respective R^2 values. (c) Same graph as shown in (b) but for temperatures ranging from 31 to 39°C. (d) Single-cell F_v/F_m values before temperature stress (= 22°C) and under selected elevated temperatures (= 31 to 39°C). Solid lines along scatterplots represent the fitting results to a normal distribution. Note the visible increase in heterogeneity at temperatures >31°C.

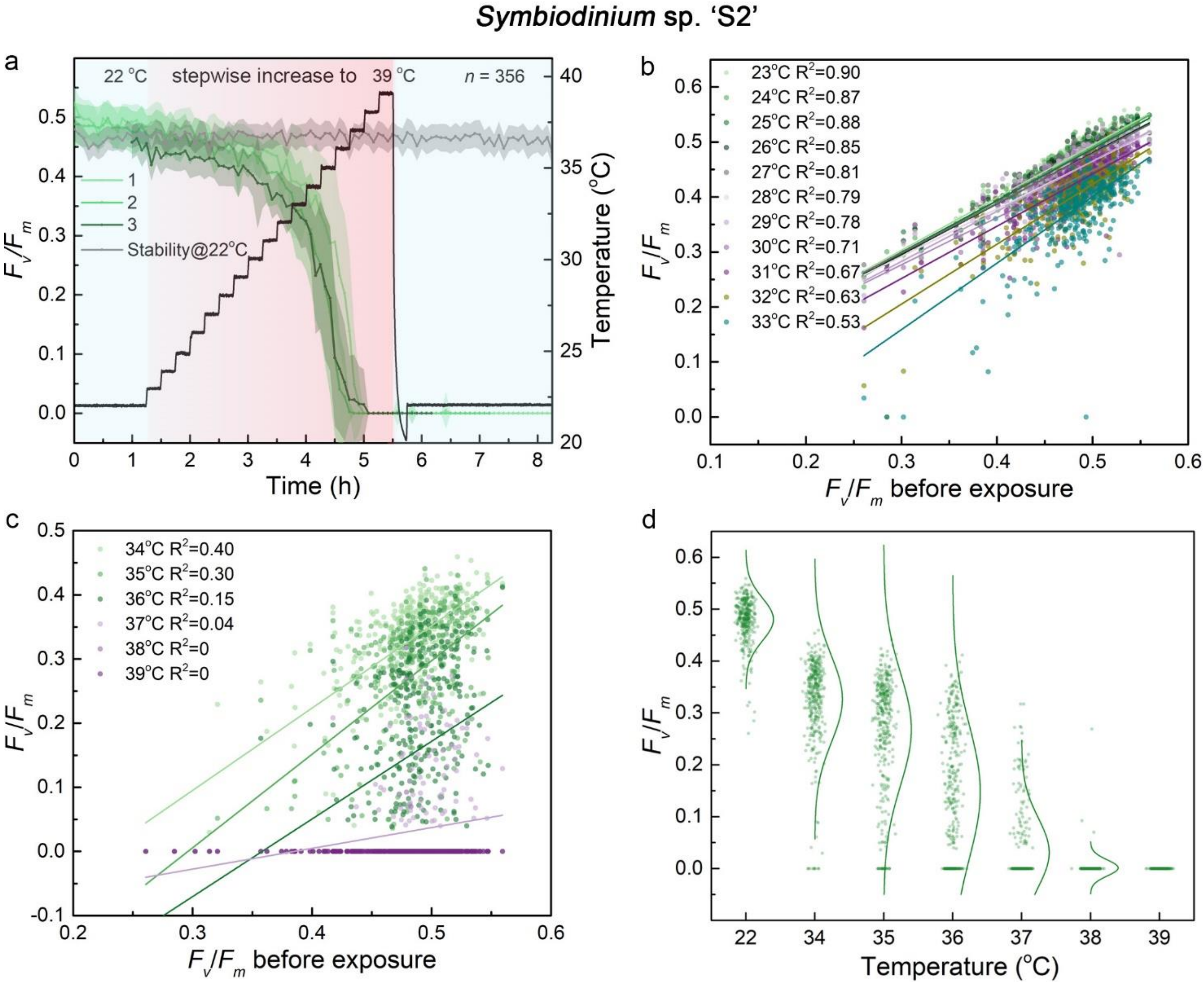

Figure S5. The photophysiological response of *Symbiodinium* sp. 'S2' single-cells to elevated temperatures. (a) The inter-experimental variability of average F_v/F_m under stepwise increasing temperatures. Average F_v/F_m values were calculated from single-cells from three independent biological experiments. These experiments (colored lines) describe the reduction in F_v/F_m under stepwise increasing temperatures (black line) and an additional experiment (gray line) that investigated the stability of F_v/F_m under repeated saturation probing but without temperature exposure.

Shaded areas represent the standard deviation in each experiment. (b) Correlations between F_v/F_m before and after temperature exposure for single-cells of *Symbiodinium* sp. ‘S2’. Here, the X-axis represents the F_v/F_m value from each individual cell before temperature exposure (at 22°C) and the Y-axis represents the F_v/F_m value from the same cell under stepwise increasing temperatures (from 23 to 33°C). F_v/F_m values at a given temperature were obtained by averaging the three F_v/F_m measurements performed at each temperature step for each cell (see supplementary materials and methods for details). Linear regression fits of the resulting scatterplots are shown together with their respective R^2 values. (c) Same graph as shown in (b) but for temperatures ranging from 34 to 39°C. (d) Single-cell F_v/F_m values before temperature stress (= 22°C) and under selected elevated temperatures (= 34 to 39°C). Solid lines along scatterplots represent the fitting results to a normal distribution. Note the visible increase in heterogeneity at temperatures >34°C.

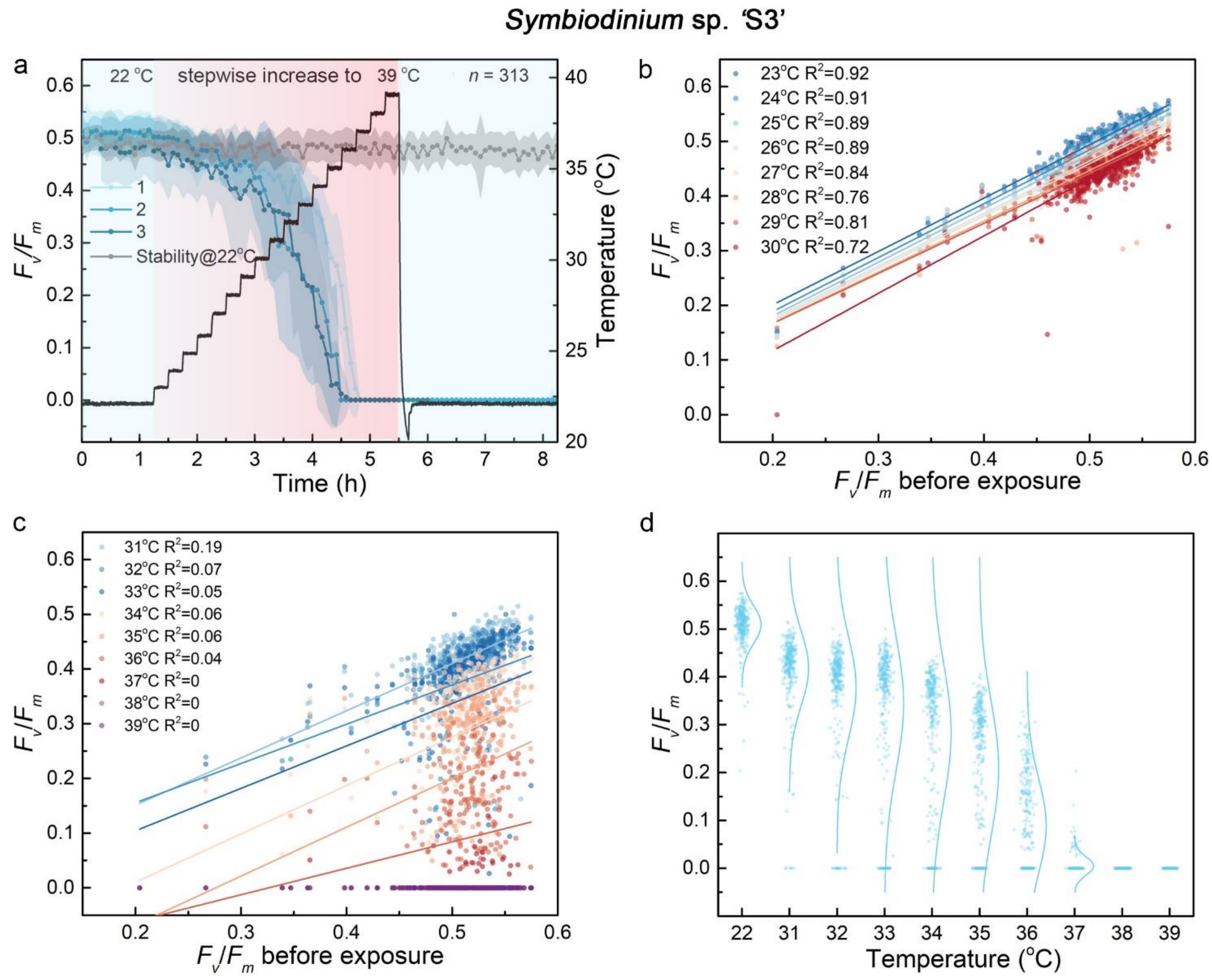

Figure S6. The photophysiological response of *Symbiodinium* sp. 'S3' single-cells to elevated temperatures. (a) The inter-experimental variability of average F_v/F_m under stepwise increasing temperatures. Average F_v/F_m values were calculated from single-cells from three independent biological experiments. These experiments (colored lines) describe the reduction in F_v/F_m under stepwise increasing temperatures (black line) and an additional experiment (gray line) that investigated the stability of F_v/F_m under repeated saturation probing but without temperature exposure. Shaded areas represent the standard deviation in each experiment. (b) Correlations between F_v/F_m before and after temperature exposure for single-cells of *Symbiodinium* sp. 'S3'. Here, the X-axis represents the F_v/F_m value from each individual cell before temperature exposure (at 22°C) and the Y-axis represents the F_v/F_m value from the same cell under stepwise increasing temperatures (from 23 to 30°C). F_v/F_m values at a given temperature were obtained by averaging the three F_v/F_m measurements performed at each temperature step for each cell (see supplementary materials and methods for details). Linear regression fits of the resulting scatterplots are shown together with their

respective R^2 values. (c) Same graph as shown in (b) but for temperatures ranging from 31 to 39°C. (d) Single-cell F_v/F_m values before temperature stress (= 22°C) and under selected elevated temperatures (= 31 to 39°C). Solid lines along scatterplots represent the fitting results to a normal distribution. Note the visible increase in heterogeneity at temperatures >31°C.

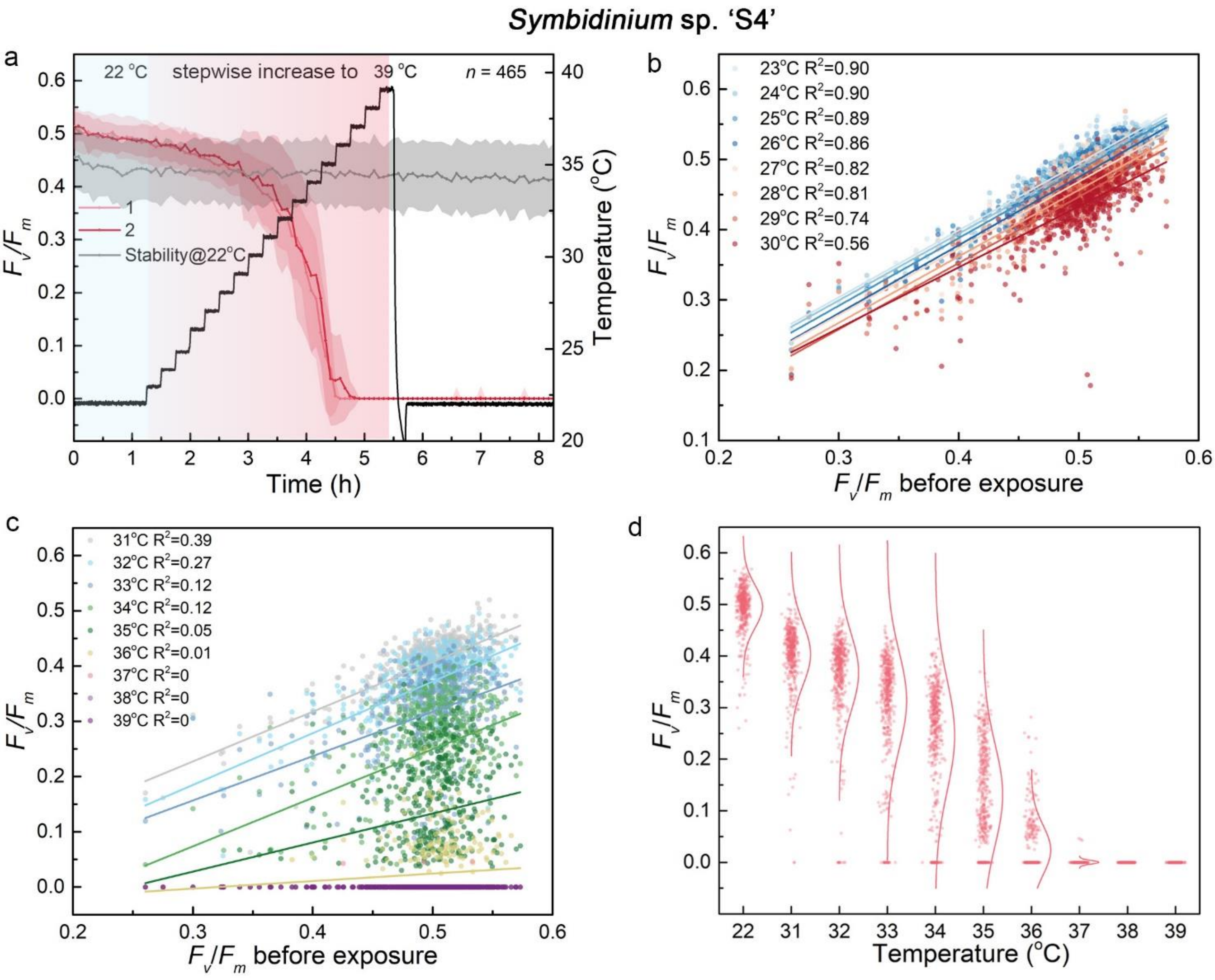

Figure S7. The photophysiological response of *Symbiodinium* sp. 'S4' single-cells to elevated temperatures. (a) The inter-experimental variability of average F_v/F_m under stepwise increasing temperatures. Average F_v/F_m values were calculated from single-cells from two independent biological experiments. These experiments (colored lines) describe the reduction in F_v/F_m under stepwise increasing temperatures (black line) and an additional experiment (gray line) that investigated the stability of F_v/F_m under repeated saturation probing but without temperature exposure. Shaded areas represent the standard deviation in each experiment. (b) Correlations between F_v/F_m

before and after temperature exposure for single-cells of *Symbiodinium* sp. ‘S4’. Here, the X-axis represents the F_v/F_m value from each individual cell before temperature exposure (at 22°C) and the Y-axis represents the F_v/F_m value from the same cell under stepwise increasing temperatures (from 23 to 30°C). F_v/F_m values at a given temperature were obtained by averaging the three F_v/F_m measurements performed at each temperature step for each cell (see supplementary materials and methods for details). Linear regression fits of the resulting scatterplots are shown together with their respective R^2 values. (c) Same graph as shown in (b) but for temperatures ranging from 31 to 39°C. (d) Single-cell F_v/F_m values before temperature stress (= 22°C) and under selected elevated temperatures (= 31 to 39°C). Solid lines along scatterplots represent the fitting results to a normal distribution. Note the visible increase in heterogeneity at temperatures >31°C.

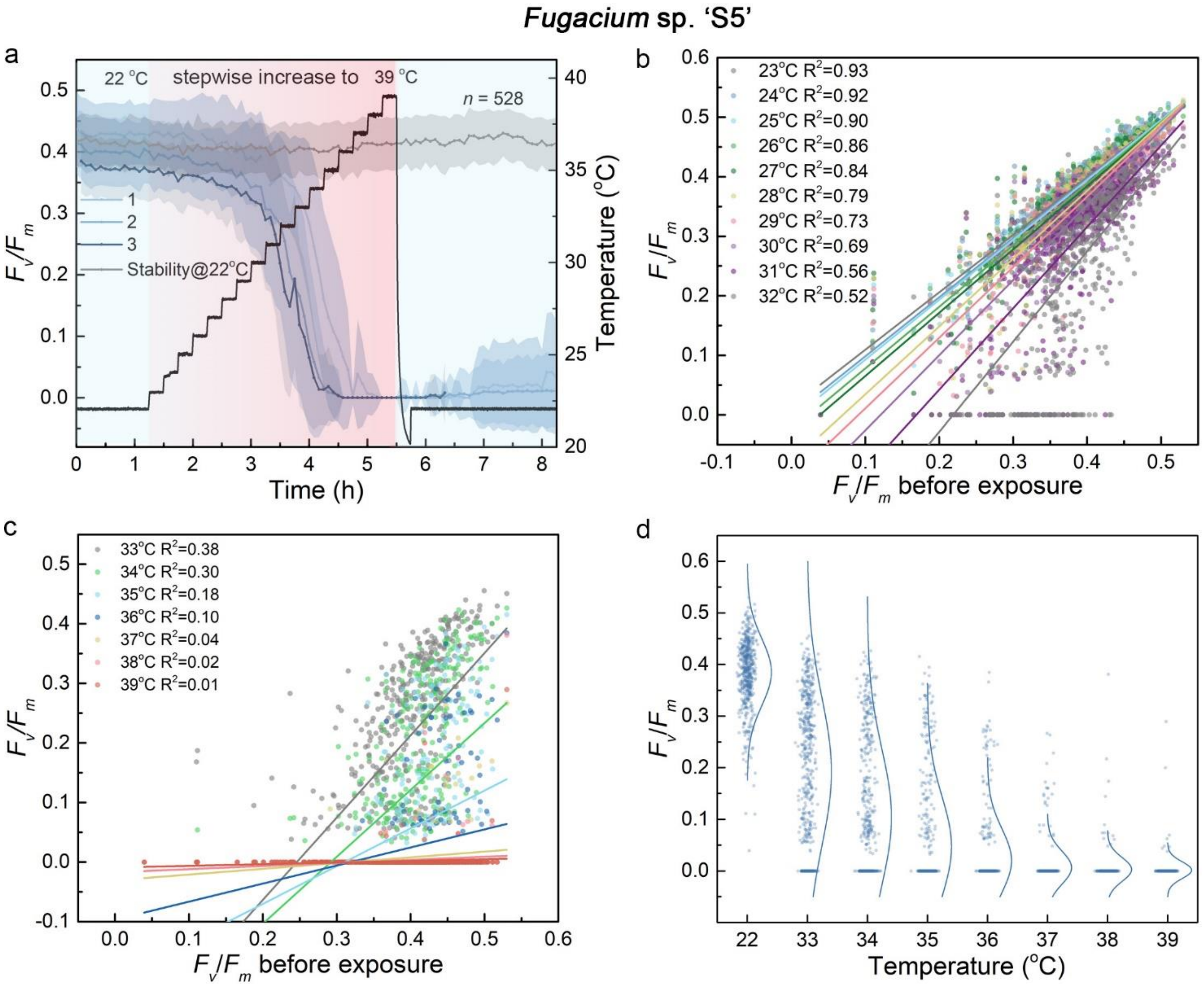

Figure S8. The photophysiological response of *Fugacium* sp. 'S5' single-cells to elevated temperatures. (a) The inter-experimental variability of average F_v/F_m under stepwise increasing temperatures. Average F_v/F_m values were calculated from single-cells from three independent biological experiments. These experiments (colored lines) describe the reduction in F_v/F_m under stepwise increasing temperatures (black line) and an additional experiment (gray line) that investigated the stability of F_v/F_m under repeated saturation probing but without temperature exposure. Shaded areas represent the standard deviation in each experiment. (b) Correlations between F_v/F_m before and after temperature exposure for single-cells of *Fugacium* sp. 'S5'. Here, the X-axis represents the F_v/F_m value from each individual cell before temperature exposure (at 22°C) and the Y-axis represents the F_v/F_m value from the same cell under stepwise increasing temperatures (from 23 to 32°C). F_v/F_m values at a given temperature were obtained by averaging the three F_v/F_m measurements performed at each temperature step for each cell (see supplementary materials and methods for details). Linear regression fits of the resulting scatterplots are shown together with their respective R^2 values. (c) Same graph as shown in (b) but for temperatures ranging from 33 to 39°C. (d) Single-cell F_v/F_m values before temperature stress (= 22°C) and under selected elevated temperatures (= 33 to 39°C). Solid lines along scatterplots represent the fitting results to a normal distribution. Note the visible increase in heterogeneity at temperatures >33°C.

References

1. Andersson, M.; Johansson, S.; Bergman, H.; Xiao, L.; Behrendt, L.; Tenje, M. A microscopy-compatible temperature regulation system for single-cell phenotype analysis – demonstrated by thermoresponse mapping of microalgae. *Lab Chip* **2021**, *21*, 1694-1705.

2. Behrendt, L.; Salek, M. M.; Trampe, E. L.; Fernandez, V. I.; Lee, K. S.; Kuhl, M.; Stocker, R. Phenochip: A single-cell phenomic platform for high-throughput photophysiological analyses of microalgae. *Sci. Adv.* **2020**, *6*, eabb2754.

3. LaJeunesse, T. C.; Parkinson, J. E.; Gabrielson, P. W.; Jeong, H. J.; Reimer, J. D.; Voolstra, C. R.; Santos, S. R. Systematic revision of symbiodiniaceae highlights the antiquity and diversity of coral

endosymbionts. *Curr. Biol.* **2018**, *28*, 2570-2580.

4. Chromaspro software version 2.1.10. https://technelysium.com.au/wp/.

5. Katoh, K.; Standley, D. M. Mafft multiple sequence alignment software version 7: Improvements in performance and usability. *Mol. Biol. Evol.* **2013**, *30*, 772-780.

6. Tan, G.; Muffato, M.; Ledergerber, C.; Herrero, J.; Goldman, N.; Gil, M.; Dessimoz, C. Current methods for automated filtering of multiple sequence alignments frequently worsen single-gene phylogenetic inference. *Syst. Biol.* **2015**, *64*, 778-791.

7. Stamatakis, A. Raxml version 8: A tool for phylogenetic analysis and post-analysis of large phylogenies. *Bioinformatics* **2014**, *30*, 1312-1313.

8. A, R. Figtree version 1.4.3. http://tree.bio.ed.ac.uk/software/figtree/ **2016**.

9. Calabrese, F.; Voloshynoyska, I.; Musat, F.; Thullner, M.; Schlomann, M.; Richnow, H. H.; Lambrecht, J.; Muller, S.; Wick, L. Y.; Musat, N.; Stryhanyuk, H. Quantitation and comparison of phenotypic heterogeneity among single cells of monoclonal microbial populations. *Front. Microbiol* **2019**, *10*, 2814.
